# Disentangling regional trade agreements, trade flows and tobacco affordability in sub-Saharan Africa

**DOI:** 10.1186/s12992-017-0305-x

**Published:** 2017-11-14

**Authors:** Adriana Appau, Jeffrey Drope, Ronald Labonté, Michal Stoklosa, Raphael Lencucha

**Affiliations:** 1 McGill University, Faculty of Medicine, School of Physical and Occupational Therapy, 3630 Promenade Sir William Osler, Montreal, QC, H3G 1Y5 Canada; 2 Economic & Health Policy Research, American Cancer Society, Inc., 250 Williams Street, Atlanta, GA 30303 USA; 3 University of Ottawa, School of Epidemiology and Public Health, 600 Peter Morand Crescent, Ottawa, ON K1G 3Z7 Canada; 4 Taxation and Health, American Cancer Society, Inc., 250 Williams Street, Atlanta, GA 30303 USA

**Keywords:** Tobacco industry, International trade, Macroeconomics, Tobacco economics, Tobacco affordability

## Abstract

**Background:**

In principle, trade and investment agreements are meant to boost economic growth. However, the removal of trade barriers and the provision of investment incentives to attract foreign direct investments may facilitate increased trade in and/or more efficient production of commodities considered harmful to health such as tobacco. We analyze existing evidence on trade and investment liberalization and its relationship to tobacco trade in Sub-Saharan African countries.

**Methods:**

We compare tobacco trading patterns to foreign direct investments made by tobacco companies. We estimate and compare changes in the Konjunkturforschungsstelle (KOF) Economic Globalization measure, relative price measure and cigarette prices.

**Results:**

Preferential regional trade agreements appear to have encouraged the consolidation of cigarette production, which has shaped trading patterns of tobacco leaf. Since 2002, British American Tobacco has invested in tobacco manufacturing facilities in Nigeria, Kenya and South Africa strategically located to serve different regions in Africa. Following this, British America Tobacco closed factories in Ghana, Rwanda, Uganda, Mauritius and Angola. At the same time, Malawi and Tanzania exported a large percentage of tobacco leaf to European countries. After 2010, there was an increase in tobacco exports from Malawi and Zambia to China, which may be a result of preferential trade agreements the EU and China have with these countries. Economic liberalization has been accompanied by greater cigarette affordability for the countries included in our analysis. However, only excise taxes and income have an effect on cigarette prices within the region.

**Conclusions:**

These results suggest that the changing economic structures of international trade and investment are likely heightening the efficiency and effectiveness of the tobacco industry. As tobacco control advocates consider supply-side tobacco control interventions, they must consider carefully the effects of these economic agreements and whether there are ways to mitigate them.

**Electronic supplementary material:**

The online version of this article (10.1186/s12992-017-0305-x) contains supplementary material, which is available to authorized users.

## Background

Open international trade is argued to be a powerful driver of economic growth and development [1]. Although trade liberalization has contributed to overall economic growth in many parts of the world, gains are often much less than proponents claim [2, 3], and the distribution of these gains has been uneven [4, 5]. In recent years, bilateral and regional trade agreements (RTAs) have become the key platform for negotiating broader and deeper trade liberalization measures. The last two decades in particular have seen a significant proliferation of RTAs, increasing from approximately 50 in 1990 to 406 as of April 2015 [6]. RTAs are considered preferential agreements as they establish, among other things, reduced tariff rates among members. Orthodox economic theory suggests that further trade liberalization through the surge of RTAs will increase trade in goods and services amongst members [7, 8]. This deepening liberalization is thought to be problematic for tobacco control [9]. Tobacco control proponents have primarily engaged with three aspects of trade liberalization as it pertains to tobacco: trade agreements and policy space, tobacco company influence and tobacco affordability [10–12].

First, there has been a proliferation of challenges to tobacco control measures through trade and investment agreements, including recent claims that plain packaging of tobacco products violates provisions within these agreements [13]. The tobacco industry have in the past used and continue to use provisions in trade and investment agreements, such as intellectual property rights protection and fair and equitable treatment provisions to stall and in some cases halt the implementation of tobacco control policies [14]. In addition, investment agreements include investor-state dispute settlement mechanisms that permit companies to directly challenge governments through international arbitration when they believe a policy violates an agreement. For instance in 2011, Philip Morris filed disputes challenging Uruguay’s (under a BIT between Switzerland and Uruguay) decision to increase the size of warning labels on tobacco packages and Australia’s (under a BIT between Australia and Hong Kong) laws on plain packaging. Philip Morris sought compensation for purported damages [15–17]. Such actions can constrain and deter government decisions to strengthen tobacco control considering the cost of litigation, particularly for smaller countries.

Second, trade and investment liberalization has given the tobacco industry an opportunity to increase its presence globally. Provisions and recommendations found in trade and investment agreements such as regulatory reviews, policy impact assessments and stakeholder consultations can further increase the tobacco industry’s influence on the policy making process and subsequently their influence on health policy decisions [18]. Research examining the industry’s efforts to undermine tobacco control in individual countries following its increased commercial presence [19–22] demonstrates that this presence likely does influence tobacco control policy and health policy in general [23–26]. This influence can also be channelled through pre-emption [27]. The tobacco industry has used trade and investment agreements to pre-empt local authority over tobacco control policies by transferring their legal challenges to an international authority like the International Centre for the Settlement of Investment Disputes or the World Trade Organization Dispute Settlement Body.

The third concern is that liberalization policies will result in reduced costs for the industry along the supply chain and greater market presence. It is feared that these cost reductions would be passed on to the consumer in the form of reduced prices, the effects of which – particularly increased consumption – could then potentially be heightened by greater promotion of these products by the tobacco industry. Research that examined the dynamics between trade liberalization in the 1980s and 1990s and the tobacco product marketplace found that under certain conditions, the elimination of tariffs increased competition leading to lower prices and higher consumption of tobacco products [28–32]. Many of these studies focused on Asian countries or former Soviet states that had state-owned tobacco monopolies that were not aggressively marketing or pricing their goods prior to liberalization, and/or were very closed to trade (i.e. high tariffs) [33–35].

In this study, we explore the relationship between trade and investment liberalization and changes in total tobacco trade in sub-Saharan African (SSA) countries. Drawing from different data sources we examine patterns of tobacco trade and investment in SSA in relation to the recent era of RTAs beginning in the late 1980s and early 1990s. To take the examination to the next level of complexity, we also explore the relationships among economic liberalization (including RTAs), tobacco trade and investment patterns, and cigarette affordability and prices. Economic theory suggests that an increase in trade liberalization and trade could lead to lower prices. This is because the reduction or removal of trade barriers reduces cost of production and can improve firm efficiency. Tobacco industry internal documents confirm the cost saving benefits of consolidating production in fewer countries in a region characterised by greater trade liberalization by taking advantage of eliminated and/or reduced trade barriers to export to other countries in the region [36–38]. There will be an observed decrease in tobacco prices if firms decide to pass savings obtained from the reduced cost of production and increased efficiency to consumers. Price is one key component of affordability, but affordability is also explained by other economic factors. Income changes (which could also stem from economic liberalization) modify the effect of price changes on demand. For example, a decrease in the price of tobacco products accompanied by a significantly larger decrease in income implies less affordability. To support our analysis on affordability, we conducted an empirical analysis to determine which factors influence cigarette prices in SSA. This statistical analysis allows us to present with greater confidence a more comprehensive picture of the relationship between trade agreements and price, and therefore to some extent, affordability. The broader emphasis on affordability then provides a more accurate picture of the relationship between product, consumer and market, even if it is not possible to predict changes in affordability directly.

Due to limited data availability for all variables of interest, we examine the relationship between trade liberalization and tobacco trade using data from 1990 to 2013 and the relationship among economic liberalization and tobacco price and affordability using data from 2007 to 2014. Although limited by data availability, our aim is to provide a defensible analysis of the complex relationship among these important variables. While we find that trade liberalization appears to have encouraged the consolidation of cigarette production, thereby shaping some trading patterns of tobacco leaf, and has been accompanied by greater cigarette affordability generally, there are multiple factors contributing concomitantly to this complex dynamic that need to be explored further. For instance, regional economic liberalization does not alone explain the changes in trade patterns, which could also realistically be partly a result of the increasing presence of leaf buying multinational companies that have reorganized global leaf supply more efficiently to meet global demand. Similarly, price is often as much a function of industry strategy and can have little to do with the most obvious micro- and macroeconomic variables.

## Methods

### Regional trade and investment agreements

**Fig. 1:**
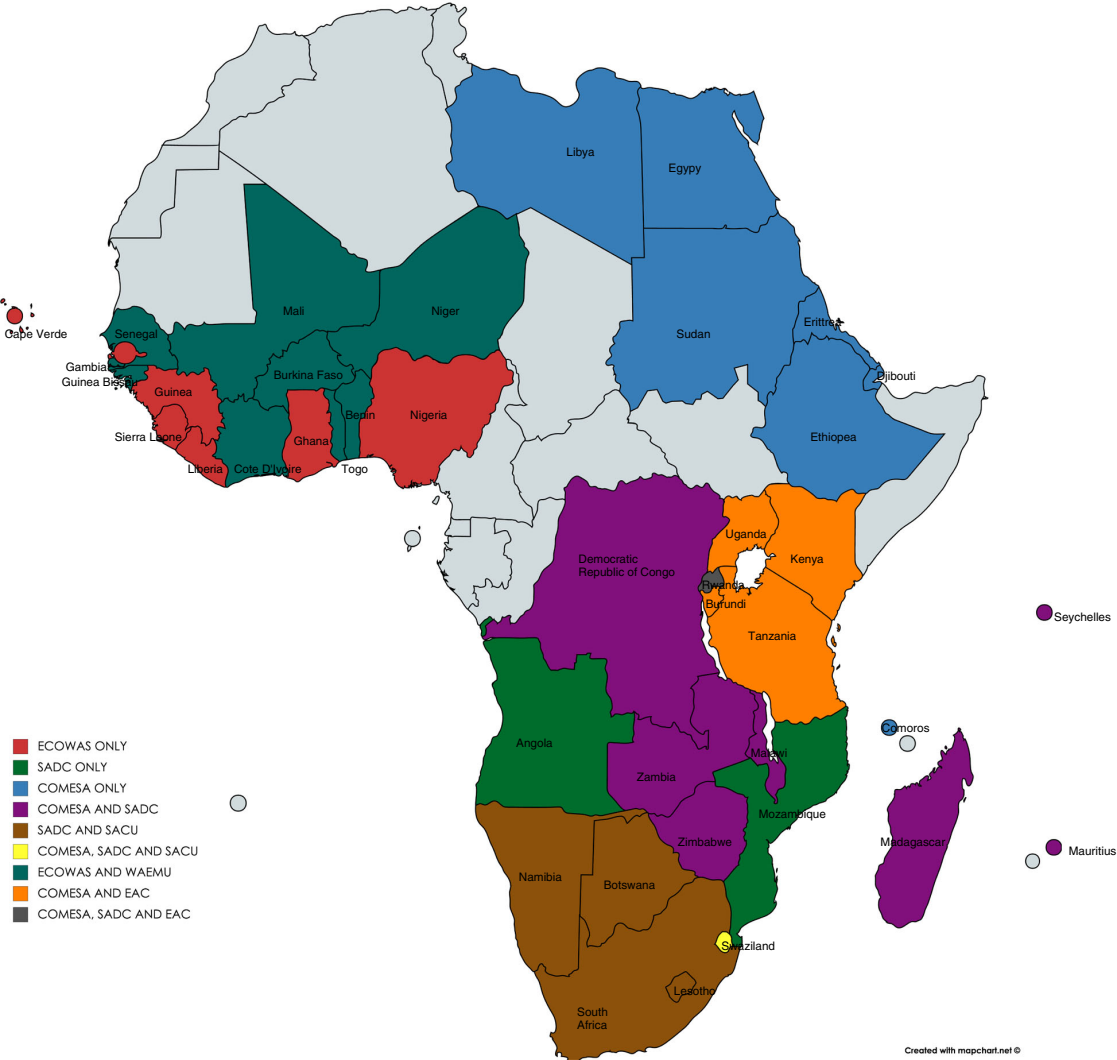
Six Major RTAs in Sub-Saharan Africa

This study focuses on the six main RTAs in sub-Saharan Africa which cover 39 out of 48 countries and have bearing on the economic policy of the member governments: the Economic Community of West African States (ECOWAS), the Common Market of Eastern and Southern African States (COMESA), the Southern African Development Community (SADC), the West African Economic and Monitory Union (WAEMU), the Southern African Customs Union (SACU) and the East African Community (EAC). There are other agreements, particularly in central Africa, but data limitations made an expanded examination unfeasible. Figure 1 shows the member countries of the six RTAs. Tobacco trade within these RTAs is subject to zero import tariffs. However, trade outside the regional agreements attracts different tariff rates depending on the country and the tobacco products. For example, according to 2013 tariff data from the International Trade Center (ITC), Kenya applies a most favoured nation (MFN)^1^ tariff of 25% on tobacco unstemmed/unstripped and an MFN tariff of 35% on cigarettes containing tobacco originating from Nigeria [39]. On the other hand, Nigeria applies an MFN tariff of 5% on tobacco unstemmed/unstripped originating from Kenya [39]. However, similar to Kenya, Nigeria applies an MFN tariff of 35% on cigarettes containing tobacco originating from Kenya [36]. In other regions, there is evidence that tobacco companies who are established in a given country or region have lobbied for these differential tariff rates to gain competitive advantage over other companies who would have to import into the region [37].

Tobacco exported from SSA countries receives preferential rates under other agreements. The 2000 Cotonou Agreement between the European Union (EU) and 77 countries from the Africa, Caribbean and Pacific Group (ACP) (which includes 48 from SSA) [6] allows partners to export tobacco to the EU with no tariffs [40]. More recently, a Duty Free Treatment for Least Developed Countries (LDCs) was signed by China and 40 countries (30 from SSA), which also offers preferential treatment on tobacco and tobacco products exported into China [6].

### Liberalization, cigarette price and affordability

Cigarette price and affordability was analyzed across the six major economic agreements mentioned above. Economic liberalization was measured by the Konjunkturforschungsstelle (KOF) Economic Globalization measure [41]. The KOF measure is an index comprising many facets of economic liberalization wherein higher values connote more liberalization; values are comparable across time and/or countries. Specifically, the KOF is comprised of i) Actual Flows (50%) [Trade (percent of GDP) (22%) Foreign Direct Investment, stocks (percent of GDP) (27%) Portfolio Investment (percent of GDP) (24%) Income Payments to Foreign Nationals (percent of GDP) (27%)] and ii) Restrictions (50%) [Hidden Import Barriers (24%) Mean Tariff Rate (28%) Taxes on International Trade (percent of current revenue) (26%) Capital Account Restrictions (23%)] [41].

We used Blecher and van Walbeek’s “relative income price” (RIP) measure to calculate affordability. The RIP is the percent of GDP per capita required to purchase 100 packs of the most popular brand of cigarettes per year [42, 43]. We also generated a regional affordability measure using a population-weighted GDP per capita measure to determine changes in affordability within the groups of countries of each economic agreement. Cigarette price data were obtained from Euromonitor from 2008 to 2014. Our analysis included price of the most sold brand in US dollars at purchasing power parity for 46 SSA countries.

To support this broader empirical perspective, we also conducted a multivariate analysis to determine which factors influence cigarette prices in SSA. In this analysis, we elect not to use affordability as the dependent (or independent) variable because affordability is itself a variable created from two variables: price and income. In demand models, the effects of those two variables are always considered separately. Using affordability as an independent variable in our analysis would mean we will have one coefficient for price and income under the assumption that price elasticity is equal to negative income elasticity, an assumption we believe is too strong for the analysis.

We adopt the model proposed by Chaloupka et al., in which cigarette prices are explained by tax rates, market concentration, and general economic conditions [44]. Cigarette prices of the most sold brand in purchasing power parity adjusted dollars (PPP$), cigarette excise tax rate, cigarette import tax rate, and the rate of other taxes applicable on cigarettes (e.g. sales tax) were obtained from the World Health Organization’s Report on the Global Tobacco Epidemic [45]. The tax rates are expressed as a percentage of price. This data covers years 2007, 2010, 2012, and 2014. The time frame for data on cigarette prices is short because widespread data collection for African countries only began in 2006. Market share information was obtained from Euromonitor and ERC market research companies [46, 47]. From both sources, reliable data on the tobacco companies’ market share were available for sixteen SSA countries: Cameroon, Côte d’Ivoire, Democratic Republic of the Congo, Ethiopia, Ghana, Kenya, Madagascar, Mauritius, Nigeria, Senegal, South Africa, Sudan, Togo, United Republic of Tanzania, Zambia, and Zimbabwe. Market share data were available only until 2010 for twelve out of these sixteen countries. For those countries, we interpolated the market share for each company in 2012 and 2014 using data from 2000 to 2010, utilizing a linear trend. Based on the market share information, we constructed the Herfindahl-Hirschman Index (HHI) of market concentration. The HHI controls for the potential influence of market structure. To capture the effect of economic conditions on tobacco prices, we also included per capita GDP in PPP-adjusted dollars from the International Monetary Fund [48]. Finally, in addition to variables proposed by Chaloupka et al., we added the KOF index to the model to capture the impacts of economic liberalization on cigarette prices. Countries’ memberships in major economic agreements are a key component of the index. We estimate a fixed-effects model in the following functional form:

$$ \mathit{\ln}{P}_{it}={\beta}_0+{\beta}_1 exc\ {tax}_{it}+{\beta}_2 imp\ {tax}_{it}+{\beta}_3 other\ {tax}_{it}+{\beta}_4\mathit{\ln}{Y}_{it}+{\beta}_5{HHI}_{it}+{\beta}_6{KOF}_{it}+{\alpha}_i+{\varepsilon}_{it} $$

where *β*_*0*_ is the intercept, *α*_*i*_ is a set of fixed constants, and *ε*_*it*_ is the error term. The dependent variable in this model is a logarithm of PPP-adjusted price in a given country (*i*) in a given year (*t*). The independent variables are cigarette excise tax (*exc tax)*, import tax (*imp tax)*, other taxes applicable on cigarettes (*other tax*), per capita GDP *(Y)*, Herfindahl-Hirschman Index *(HHI),* and the Konjunkturforschungsstelle *(KOF).*

### Tobacco trade and investment data sources

Tobacco trade data from 1990 to 2013 were obtained from the United Nations COMTRADE database and the WTO Trade Analysis and Information System.^2^ Tobacco investment data were obtained from an exhaustive survey of multiple sources, including published local news accessed online, tobacco companies’ investment reports, and government investment reports and publications. Data on bilateral investment treaties and trade agreements were obtained from the International Centre for Settlement of Investment Disputes (ICSID) Database of Investment Treaties and WTO Preferential Trade Arrangements Database respectively. Because many African countries fail to report trade flows and trade barriers, missing data are a major challenge in conducting empirical analysis of trade-related impacts. We combine our best available data sources in order to construct a defensible relationship between trade and investment agreements and tobacco trade flows.

## Results and discussion

### Tobacco industry investments

**Fig. 2:**
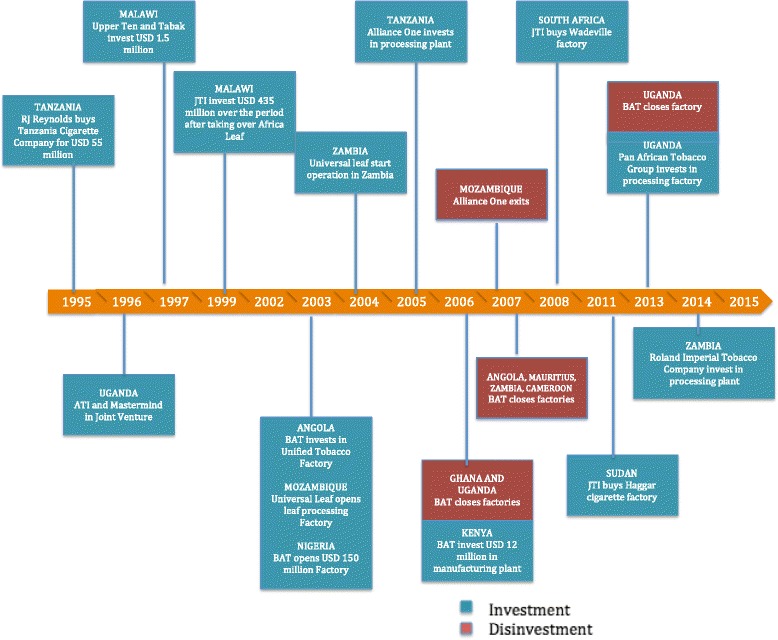
Major Tobacco Industry Investments And Disinvestments In Sub-Saharan Africa

Major investment activities by the tobacco industry are reported in Fig. 2. Investments by tobacco companies within SSA have taken the form of acquisitions of state-owned factories, joint ventures (3.1.1), and establishing manufacturing facilities (3.1.2) many contributing to broader regional consolidation of the sector (3.1.3).

#### Acquisition of State-Owned Companies and Domestic Companies

Prior to liberalization, most African national governments maintained sole ownership of companies. In the era of economic liberalization, countries opened their economy to the private sector. In some cases, this gave transnational tobacco companies (TTCs) the opportunity to purchase state-owned tobacco companies, including leaf processing and cigarette making. In 1995, for instance, RJ Reynolds purchased the state-owned Tanzania Cigarette Company (though subsequently Japan Tobacco International acquired a 75% stake in the company) [49]. Other TTCs made similar acquisitions of local or regional tobacco companies to expand their market, including the purchases of the Africa Leaf Company in Malawi in 1999 and Haggar Cigarette in Sudan in 2011, both by JTI [50, 51]. Although these decisions do not appear directly related to RTAs, they were likely enabled by trade and investment liberalization requirements associated with IMF structural adjustment loans and economic reform programmes. In the early 1990s, an era when most developing countries were seeking economic growth/debt repayment strategies, the IMF encouraged the privatization of state-owned companies under the premise that privatization would lead to an increase in competition and a decrease in costs of production due to higher efficiency, thereby engendering revenue generation [52]. Specifically for tobacco, the IMF asserted that privatizing state-owned tobacco companies would create an environment where governments would be more likely to impose stronger tobacco control measures [53]. Evidence from the Former Soviet Union demonstrates that privatization was followed by aggressive marketing, a shift in trade patterns, lower tobacco prices, an increased influence on tobacco control policies to favour transnational tobacco companies and subsequently increased consumption of tobacco products [29, 52].

#### Building tobacco manufacturing factories

The TTCs have also invested in new manufacturing plants or the expansion of existing ones to increase production capacity. In 1997, Upper Ten and Tabak invested approximately USD 1.49 million in Malawi [54]. Similarly, in 2003, BAT commissioned a manufacturing plant in Nigeria, one of the largest investments by a tobacco company in SSA at a value of $150 million [55]. Universal Leaf Africa opened new factories in Nigeria and Mozambique respectively [55, 56]. JTI also acquired the Wadeville factory in South Africa [57]. More recently, in 2013, Pan African Tobacco group invested in a tobacco-processing factory in Uganda [58]. Reports also suggest that the Chinese government’s parastatal, the Chinese National Tobacco Corporation (CNTC), has invested in the tobacco industry in Zimbabwe in the last 5 years [59]. In 2007, the two governments signed a financial agreement in the form of a USD 58 million loan from China for the purchase of agricultural farm equipment and tools. In return for this loan, Zimbabwe was to deliver 110,000 t of tobacco for two years to the CNTC [60].

#### Regional consolidation

In addition to investing, some TTCs have rationalized their operations by consolidating manufacturing into fewer countries within SSA. For instance, BAT invested approximately USD 12 million to increase manufacturing in a plant in Kenya after closing their factories in Uganda and Rwanda in 2006 and Mauritius in 2007 [61, 62]. While production cost reductions were part of the logic, it is important to note that Kenya, Rwanda, Uganda and Mauritius are all members of COMESA, while Kenya, Rwanda and Uganda are members of the EAC. Preferential trade agreements appear to have shaped this consolidation, which, in turn, has shaped trading patterns of tobacco leaf within the region. For example, BAT invested heavily into the Nairobi tobacco leaf processing and cigarette manufacturing plants, which use both domestic leaf and imports from countries in the COMESA and EAC agreements. Similarly, in 2006 the BAT factory in Ghana was closed and regional manufacturing moved to Nigeria; both countries are members of ECOWAS [63]. In addition, BAT upgraded and modernized its processing and manufacturing center in South Africa to serve as the southern African production hub. Following this move, they closed their factories in Angola in 2007 and Zambia in 2006, all three countries being members of SADC [63, 64]. This consolidation has created BAT regional hubs for West Africa (Nigeria), East Africa (Kenya) and Southern Africa (South Africa) that serve as regional buying hubs for tobacco leaf and distribution of manufactured products. Similar rationalization projects have been implemented within regional agreements by TTC in other regions as far back as the 1980s. In Central America for example, BAT company documents show that it lobbied for higher external tariffs for products imported into the Central American Common Market but lower internal tariffs for trade within the Common Market [37]. Following this lobbying effort, BAT embarked on a rationalization project in which they closed their factories in Guatemala, El Salvador, Nicaragua and Costa Rica, consolidating production in Honduras [38]. Consolidation allowed BAT to produce in Honduras and export to the other members, taking advantage of the lower internal custom duties to significantly reduce cost and gain competitive advantage against other TTCs [37].

### Trade patterns

Tobacco exports data from COMTRADE for SSA were compiled and examined to identify trade patterns. The results presented in this section are for major tobacco leaf-producing and/or tobacco manufacturing countries that have demonstrated significant changes in trade patterns over the last two decades. Additional file 1: Figure S1, Additional file 2: Figure S2, Additional file 3: Figure S3, Additional file 4: Figure S4, Additional file 5: Figure S5, Additional file 6: Figure S6, Additional file 7: Figure S7 and Additional file 8: Figure S8 present graphical representations of tobacco exports from Malawi, Kenya, Zambia, Zimbabwe, Tanzania and Uganda. A large percentage of Malawi’s leaf exports go to European countries (Additional file 1: Figure S1), notably Germany, France, Switzerland, Belgium and Denmark. After a dramatic increase from 2005 to 08, there was a reasonably steep decline after 2008 accompanied by a decline in tobacco use, perhaps subsequent to the financial crisis, although there has been a recent uptick again [65]. This dynamic strongly suggests that Malawi is thoroughly incorporated into a global tobacco leaf supply chain and much less a regional one. Beyond high volumes of Malawian tobacco exports to Europe, there are some other important trends. For instance, Malawi’s trade within Africa has been uneven, but has also remained minor compared to Europe. There has been, however, a notable increase in exports to Asia, mainly China, beginning as early as 2006.

Tobacco leaf exports from Kenya (Additional file 2: Figure S2) present a more complex scenario. Like Malawi, tobacco leaf exports from Kenya to Europe have increased steadily since the mid-1990s. But since the time of BAT’s consolidation into Kenya’s regional tobacco manufacturing hub, there has been an overall decrease in leaf exports in general to the three traditional regional destinations of Africa, Asia and the Americas. For example, while there was a large increase in leaf exports to African neighbours peaking in 2011, there was then a precipitous drop in regional exports. Similarly, leaf exports to Asia expanded rapidly until 2009 but decreased noticeably thereafter. Trade to the Americas has been uneven, though usually insignificant, with anomalies most likely reflected ephemeral global demand for the types of tobacco leaf that Kenya was producing in a particular year. It appears that Kenya is retaining much of its tobacco leaf for domestic manufacturing purposes (Additional file 3: Figure S3).

While Kenya was a very small exporter of manufactured tobacco products prior to 2005, producing mainly for domestic consumption, the country’s tobacco products exports to other African countries skyrocketed thereafter. The principal destinations for these products were Mauritius, Rwanda and Uganda (Additional file 4: Figure S4), all countries where BAT had closed manufacturing operations. These results capture the design of BAT’s consolidation strategy, which appears to capitalize on the fact that these four countries belong to either the EAC or COMESA. By consolidating manufacturing in Kenya, BAT has access to preferential treatment with these countries. We found a similar dynamic in ECOWAS particularly with Nigeria and Ghana, wherein BAT closed its cigarette factory in Ghana and expanded manufacturing operations in Nigeria. Though not shown here, tobacco leaf exports from Ghana to Nigeria increased markedly with a corresponding increase in cigarette exports from Nigeria to Ghana. Although this study does not directly test the influence of tobacco tariffs on FDI investments by tobacco companies, a BAT document supports the interpretation that tobacco companies have strategically located manufacturing and processing factories in countries that have access to preferential tobacco tariffs (and/or favourable tobacco tax structures) to be able to export at a lower cost [36, 37].

For Zambia, the trends are slightly more complex. There is a clear trend toward more intra-African trade (Additional file 6: Fig. S6). Since BAT’s Zambian manufacturing operations closed, a significant amount of the Zambian tobacco leaf goes to its African neighbour, Malawi. With tobacco, a relatively new large scale crop in Zambia, there is not yet sufficient infrastructure to process it, while this capacity exists in Malawi. As noted earlier, a large proportion of the tobacco leaf exported from Malawi goes to Europe and China, which likely means the same for Zambian tobacco that transits through Malawi. The leaf exports from these countries to the EU benefits directly from the Cotonou Agreements, which provide tariff-free access to the EU market. In terms of Zambia’s direct tobacco leaf exports, there is also a marked increase to Asia, with more exports to Asia than to Europe, although fewer than to its neighbours in Africa. Most of Zambia’s direct tobacco leaf exports are destined for China. The increases in tobacco exports after 2010 from countries such as Malawi and Zambia to China came after China’s Duty Free Treatment to LDCs came into force. Notably, there has been a steady decline in exports to South Africa, which had once been a major importer of Zambian tobacco.

Between 1997 and 2010, the top export destinations for Tanzanian tobacco – like Malawi’s – have also been in Europe, including Belgium, Germany, the Netherlands and Switzerland (Additional file 8: Figure S8).

There are other factors that can potentially contribute to changes in tobacco trade such as the implementation of the Framework Convention on Tobacco Control (FCTC). The FCTC is a WHO public health treaty that came into force in 2005 establishing key tobacco control measures to be implemented by member states. Although the ratification of the FCTC and corresponding implementation of its provisions will affect price, and in principle would limit tobacco supply, we do not anticipate that ratification of the FCTC by countries within SSA played a role in the changes we observe in trade for two main reasons. First, although 37 out of 47 countries in SSA had ratified the FCTC (Eritrea, Malawi, South Sudan and Zimbabwe had neither signed nor ratified the FCTC) as of 2013 (the end of our study period), most of these countries ratified the FCTC between 2004 and 2009. However, most African countries have not implemented the FCTC provisions into national law. In a recent study on the implementation status of the FCTC in SSA, as of 2014, only 9 out of 23 countries with available reports had achieved above 50% implementation rates for demand side tobacco control policies [66]. For example, Kenya, one of the leaders in tobacco control in SSA, has only recently amended its tobacco tax structure (2011–2015) to align with the FCTC provisions [67]. Out of these 9 countries that had implemented some provisions of the FCTC, only 2 have significant trade in tobacco [66]. Second, there is evidence from some countries indicating that the FCTC plays little role in shaping investment and trade policies in SSA. For example, evidence from Zambia collected in 2014 (6 years after the country ratified the FCTC) indicates that the economic sector continues to induce tobacco supply by providing investment incentives for tobacco manufacturing [68].

These results align with the evidence that the tobacco industry has increased its presence in Africa and other emerging tobacco markets by capitalizing on developing country’s economic dependence on tobacco and weaker tobacco control commitments to increase FDI susbstantially [69–71]. Furthermore, cheap labor in tobacco farming and reduced trade barriers as a result of trade liberalization has allowed the industry to efficienlty export globally from SSA while reaping high profits.

### Economic liberalization, cigarette price and affordability

To what extent does the proliferation of RTAs and the increase in leaf trade correspond to cigarette prices and affordability in the region? Table 1 presents aggregate regional changes in economic liberalization, cigarette prices and cigarette affordability.

**Table 1:** Regional changes in economic liberalization, prices and cigarette affordability

Regional Agreement	[1] Average KOF – Economic liberalization 1987 vs. 2012	[2] International dollars at purchasing power Parity 2008	[3] International dollars at purchasing power Parity 2010	[4] International dollars at purchasing power Parity 2012	[5] International dollars at purchasing power Parity 2014	[6] Annual change in affordability 2006–2012* (Δ% of GDP per cap)
COMESA	28.4–44.0	3.27	3.21	3.27	3.62	−0.96
SADC	44.9–57.7	4.02	4.57	4.60	4.99	−0.21
ECOWAS	34.7–47.8	1.92	1.87	1.84	2.16	−0.97
WAEMU	31.8–42.9	1.93	1.89	1.85	1.99	0.11
SACU	59.4–63.7	5.07	5.25	5.62	6.24	−0.27
EAC	19.2–39.0	2.06	2.52	2.31	2.65	−1.49

First, there has been a clear and marked shift toward greater economic liberalization across the continent. As column 1 of Table 1 illustrates, the countries in these six major African economic agreements all demonstrate economic liberalization in their regional aggregates, as measured by the KOF Economic Globalization measure. SACU experienced the smallest change but was clearly the most liberalized region at the starting point in the late 1980s, while the EAC experienced the largest shift but remains the least liberalized by the KOF measure.

Second, columns two to five show the price of the most sold brand in US dollars at purchasing power parity for four years: 2008, 2010, 2012 and 2014 obtained from Euromonitor. On average, there have been price increases in each regional agreement, although some regional agreements experienced price decreases in certain years. For instance, in ECOWAS there was a 2% decrease in price between 2008 and 2010, and between 2010 and 2012; however there was an 18% average price increase between 2012 and 2014. This is also seen in WAEMU, which is unsurprising considering the membership overlap with ECOWAS. It is worth noting that in both agreements there is currently a tax ceiling on tobacco products, which may be keeping prices lower than in other regions. COMESA experienced a 1.8% decrease in price for the years 2008 to 2010. The EAC experienced an 8.18% decrease in prices between 2010 and 2012. It is important to note that out of the 46 countries included in this analysis, for 10 countries prices did not change meaningfully.

Last, as column 6 illustrates, five of the six regions demonstrate greater affordability of cigarettes, much as economic theory on trade liberalization might predict. The EAC demonstrates the most dramatic change in affordability over time, dropping from approximately 17% of GDP per capita to buy 100 packs of cigarettes to 15.5%. Only in WAEMU did cigarettes become less affordable, but only very slightly at −0.11% of GDP per capita.

It is important to note that there is variation in cigarette affordability in SSA and that this preliminary analysis cannot fully explain this variation. It is possible that some of these broader trends in affordability are due to economic liberalization, but this relationship requires further research to identify the precise determinants. In addition to economic liberalization, the level of excise taxes imposed on tobacco products resulting from the implementation of the FCTC will affect affordability by increasing cigarette retail prices. As noted above, it is not just price but also income growth that drives affordability, and parts of SSA enjoyed sustained and reasonably high growth during the period under examination. Although SSA remains highly unequal, it did reduce its Gini index (a measure of income inequality) by, on average, almost 5 points since 1990 [72], implying slightly more purchasing power for a large portion of the population. Following the broader logic that economic liberalization leads to economic growth, it may be that some of these effects on affordability are occurring through this more indirect mechanism – i.e. reaping some broader economics rewards of overall liberalization – not just through liberalization specific to the tobacco sector.

Table 2 summarizes our findings from the multivariate analysis. The only factors that are significantly associated with cigarette prices in our sample were cigarette excise taxes and per capita GDP.^3^ Specifically, an increase in excise tax rate by one percentage point was associated with cigarette price increase of 1.1%. Additionally, a 1% increase in per capita GDP was associated with a 0.6% increase in cigarette prices. Other variables were not significant in the model. Our findings are consistent with a substantial body of evidence from other parts of the world [73]. The finding that excise taxes and per capita GDP and not import taxes and other taxes applied on cigarettes have a significant impact on cigarette prices is not surprising. Excise taxes are particularly effective because they are imposed specifically on cigarettes. Because excise taxes are targeted to particular products, any change in cigarette excise tax rate is more likely to influence the price of cigarettes relative to prices of other goods and services in the economy. In contrast, reductions in import taxes typically involve larger groups of products. Moreover, import taxes do not affect domestically-produced goods. Therefore, with a change in import tax rates, the shift in relative cigarette price is likely to be smaller than a change in the excise tax rate.

**Table 2:** Determinants of cigarette prices in Sub-Saharan Africa

Variables	Coefficient Estimates	Standard Error
Excise tax	0.011*	0.005
Import tax	0.008	0.023
Other taxes	−0.025	0.014
Logarithm of per capita GDP	0.617**	0.189
Market Concentration (HHI)	−0.551	0.558
Economic Liberalization (KOF)	−0.025	0.016
Constant	−2.630	1.492

*N* = 64; **p < 0.01; *p < 0.05

Furthermore, with higher incomes, people can afford more goods and services. Studies indicate that the income elasticity of cigarette demand is generally higher in low- and middle-income countries (LMICs) than in high-income countries [73], which suggests that LMICs observe particularly large increases in the demand for cigarettes resulting from income growth. Tobacco companies take advantage of this fact, and increase cigarette prices to boost their profits. These price increases are, however, not large enough to outpace the income growth rate. On average, a 1% increase in per capita GDP in our sample was associated with only a 0.6% increase in cigarette prices, which suggests that cigarettes became more affordable over time, as income increased.

## Limitations

It is important to note that there is considerable country-level variation in the aggregate findings presented and discussed above. Subsequent studies should seek to buttress these findings with country-specific analyses. Space constraints prevent us from this kind of country-level analysis. This research has also been constrained due to a lack of comprehensive and reliable data on trade flows and trade barriers in Africa. The COMTRADE data are far from perfect but are reliable enough to present trends in direction of change accurately and magnitude of change adequately. Also, our price data do not reflect illicit products and over-privilege urban retail outlets. If better data become available, researchers should use time-series econometric analysis to determine the impact over time of RTAs, FDI and other trade barriers on tobacco trade in sub-Saharan Africa. Similarly, in the absence of official tobacco-specific FDI data, we relied on multiple sources to obtain data on tobacco investments, limiting the comprehensiveness of the tobacco-specific FDI data.

## Conclusion

This study explored the associations among economic liberalization, regional trade agreements, tobacco trade and cigarette price and affordability in sub-Saharan Africa. Major economic agreements appear to shape the patterns of tobacco trade and investment in the region, though the evidence demonstrates that the causal pathways are complex. In short, the increasingly liberalized global economic system gives tobacco firms more options in terms of how they source their tobacco leaf. There are at least two relevant facets to the larger supply chain needs of the tobacco industry. The first is related to leaf varietal. Some cigarettes have characterizing flavours and require specific leaf to meet a particular market’s flavour-related preferences. For example, Malawi exports most of its harvest to Europe, and increasingly to China, which have sizeable demand for the specific type of Burley tobacco that Malawi produces. The second is related to where the specific firms operate. It is clear that BAT has a specific African strategy to produce in regional hubs and to source much of the tobacco leaf from the region. But other global firms also want the high quality and affordable leaf, and leaf-buying multinationals like Alliance One and Universal Leaf operate widely on the continent and help to fulfill this global demand.

Findings from our multivariate analysis suggest that trade and investment agreements likely only have a small or moderate impact on cigarette prices in SSA. Import duties, market concentration, and trade and investment liberalization probably lie on a causal path between RTAs and cigarette prices, but the coefficients for these variables were not statistically significant in our analysis. The only significant variable that might be modified by RTAs is income. This suggests that trade liberalization that leads to higher per capita income will result in higher cigarette prices. This effect is, however, modest, because many factors outside trade liberalization influence countries’ economic conditions, and because our estimated coefficient suggests that these price increases are not large enough to make cigarettes less affordable.

In conclusion, trade and investment agreements do shape firm behaviour and trade patterns leading to efficiency gains, however these agreements do not seem to have any large, systematic effects on price and/or affordability. Efficiencies realized by the tobacco industry from trade and investment liberalization do not translate directly to increased affordability but are almost certainly increasing profits for these firms. For instance, according to the BAT Annual Reports, Africa and Middle East region saw an 18.5% and 17% increase in profits in 2010 and 2011 respectively, which was as a result of growth in volume and market share in countries such as South Africa, Nigeria and Egypt [74, 75]. Future research should continue to elucidate the exact pathways through which trade liberalization might shape the market for tobacco products. In light of this complex reality, and based on our analysis, it is clear that excise tax measures remain a crucial strategy to increase cigarette prices and reduce affordability. The tobacco industry, facilitated by trade agreements, has increased its presence in SSA. Empirical research has shown that this has been associated by increased interference in policy making through intensive lobbying of government officials and stakeholders, pre-emption and manipulation of proposed laws to stall implementation of tobacco control measures [18, 27, 71]. It will also be important to examine how trade agreements may shape the policy environment to entrench tobacco production in SSA. The latter is particularly important as tobacco control proponents engage with supply side issues.

## Additional files

Additional file 1: Figure S1. Malawi Tobacco Leaf Exports (IN 1000 USD). (PDF 132 kb)

Additional file 2: Figure S2. Kenya Tobacco Leaf Exports (IN 1000 USD). (PDF 125 kb)

Additional file 3: Figure S3. Kenya Manufactured Tobacco Product Exports To Africa (IN 1000 USD). (PDF 125 kb)

Additional file 4: Figure S4. Kenya Manufactured Tobacco Products To Uganda, Rwanda And Mauritius (IN 1000 USD) (PDF 150 kb)

Additional file 5: Figure S5. Uganda Export Destination. (PDF 107 kb)

Additional file 6: Figure S6. Zambia Export Destination. (PDF 123 kb)

Additional file 7: Figure S7. Zimbabwe Export Destination. (PDF 116 kb)

Additional file 8: Figure S8. Tanzania Export Destination. (PDF 124 kb)

## Abbreviations

ACP: Africa, Caribbean and Pacific Group
BITs: Bilateral Investment Treaties
CACM: Central Africa Common Market
CNTC: Chinese National Tobacco Corporation
COMESA: Common Market of Eastern and Southern African States
EAC: East African Community
ECOWAS: Economic Community of West African States
EU: European Union
FCTC: Framework Convention on Tobacco Control
ICSID: International Centre for Settlement of Investment Disputes
ITC: International Trade Center
KOF: Konjunkturforschungsstelle
LDCs: Least Developed Countries
MFN: Most Favoured Nation
RIP: Relative Income Price
RTAs: Regional Trade Agreements
SACU: Southern African Customs Union
SADC: Southern African Development Community
SSA: Sub-Saharan African
TTCs: Transnational Tobacco Companies
WAEMU: West African Economic and Monitory Union

## Footnotes

1 MFN refers to the treatment of all trade partners equally by providing trading partners with non-discriminatory terms of trade.

2 Harmonized System Codes 24, 2401 and 2402 was used to compile tobacco trade data.

3 In a separate analysis, we control for the effect of ratification of the FCTC and implementation of the FCTC overtime but do not find a significant effect of these two variables on cigarette prices as anticipated. The results are not presented in this paper.
