# Supplementary figures and images for: Disentangling regional trade agreements, trade flows and tobacco affordability in sub-Saharan Africa
Source: Global Health. 2017 Nov 14;13:81. doi: 10.1186/s12992-017-0305-x (PMC5686832; doi:10.1186/s12992-017-0305-x)

Supplementary Figure 1: Malawi Tobacco Leaf Exports (IN 1000 USD)

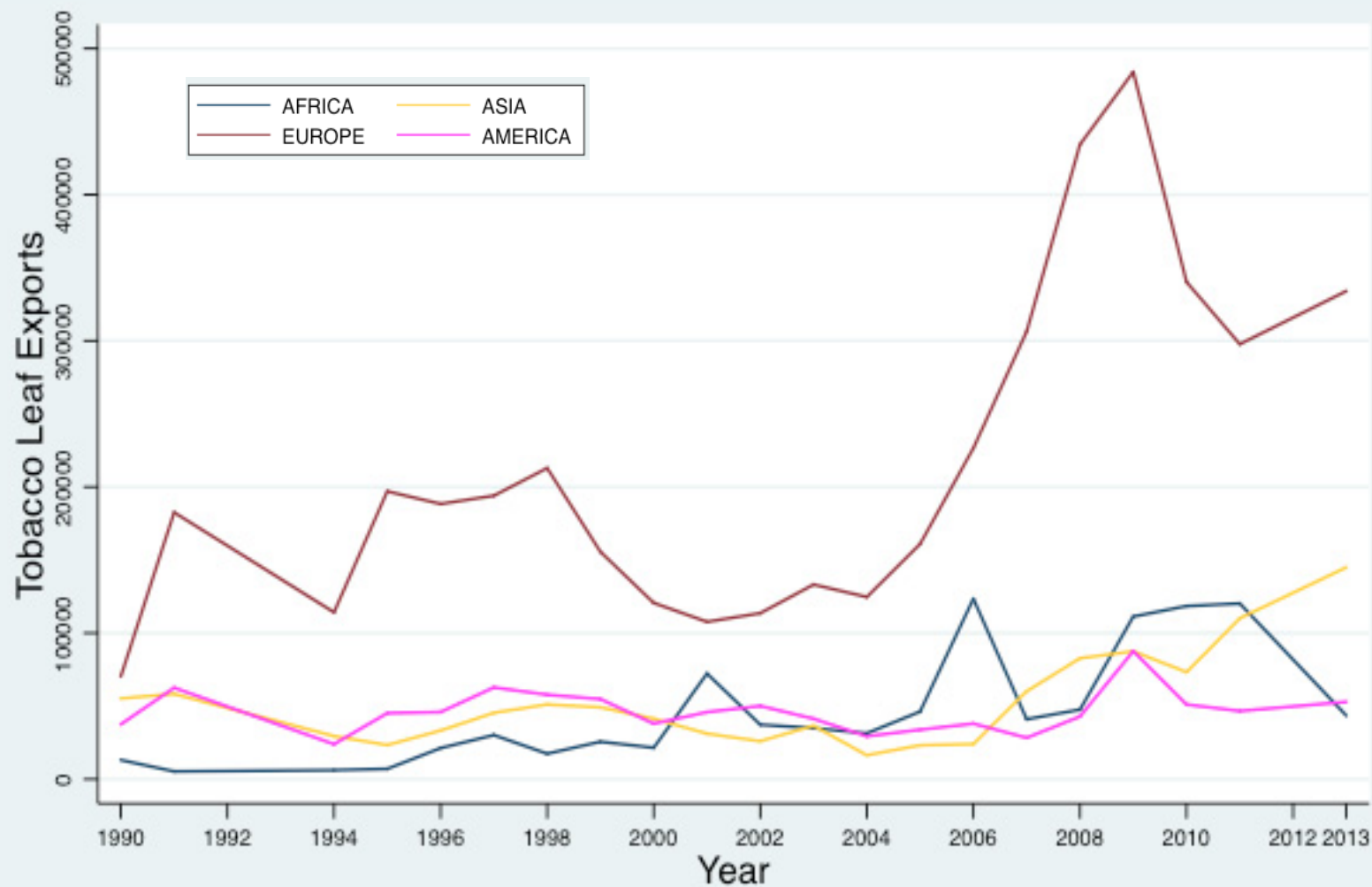

Supplement: Supplementary file 1 — Malawi Tobacco Leaf Exports (IN 1000 USD). (PDF 132 kb) [file 12992_2017_305_MOESM1_ESM.pdf]

Supplementary Figure 2: Kenya Tobacco Leaf Exports (IN 1000 USD)

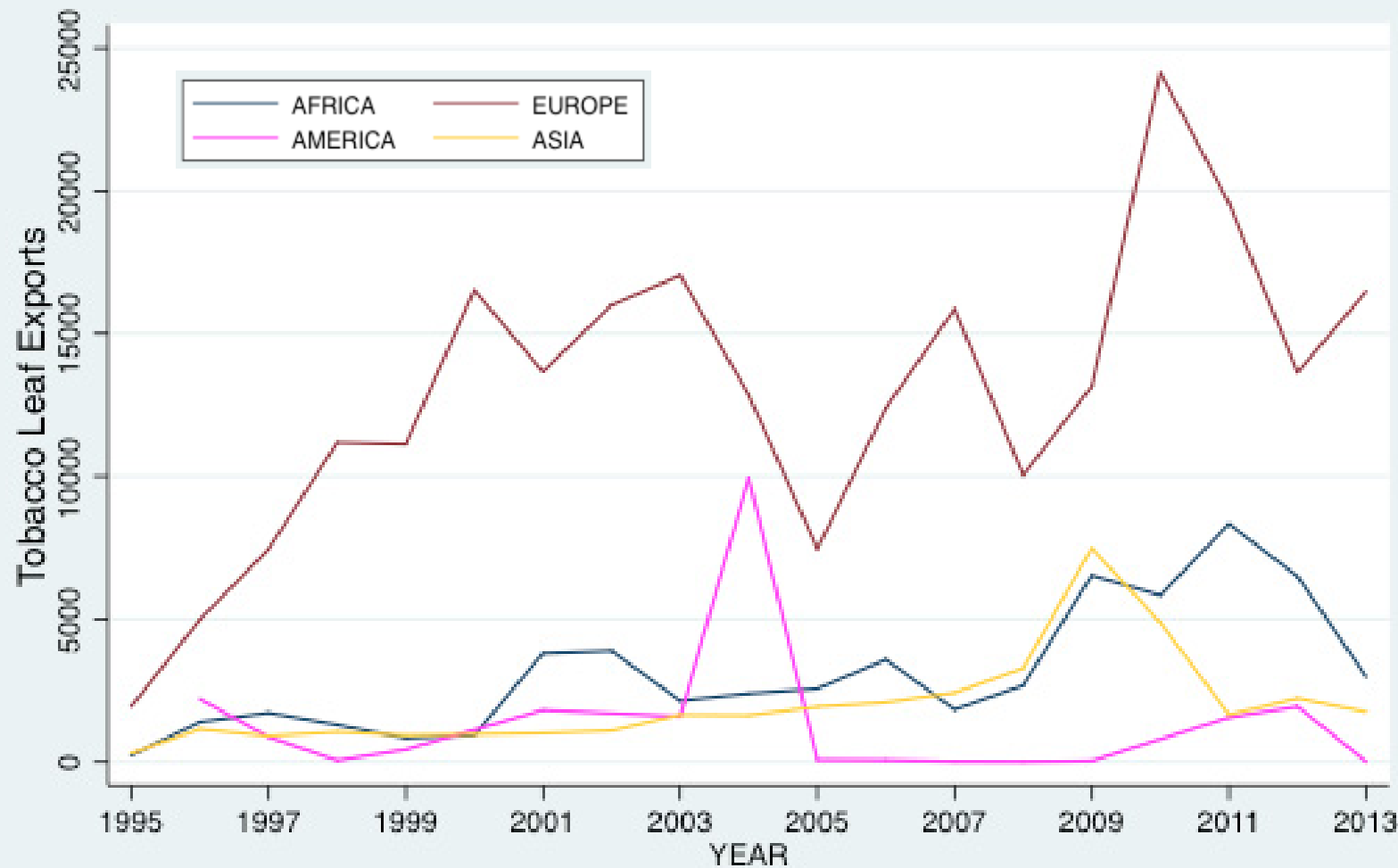

Supplement: Supplementary file 2 — Kenya Tobacco Leaf Exports (IN 1000 USD). (PDF 125 kb) [file 12992_2017_305_MOESM2_ESM.pdf]

Supplementary Figure 3: Kenya Manufactured Tobacco Product Exports To Africa (IN 1000USD)

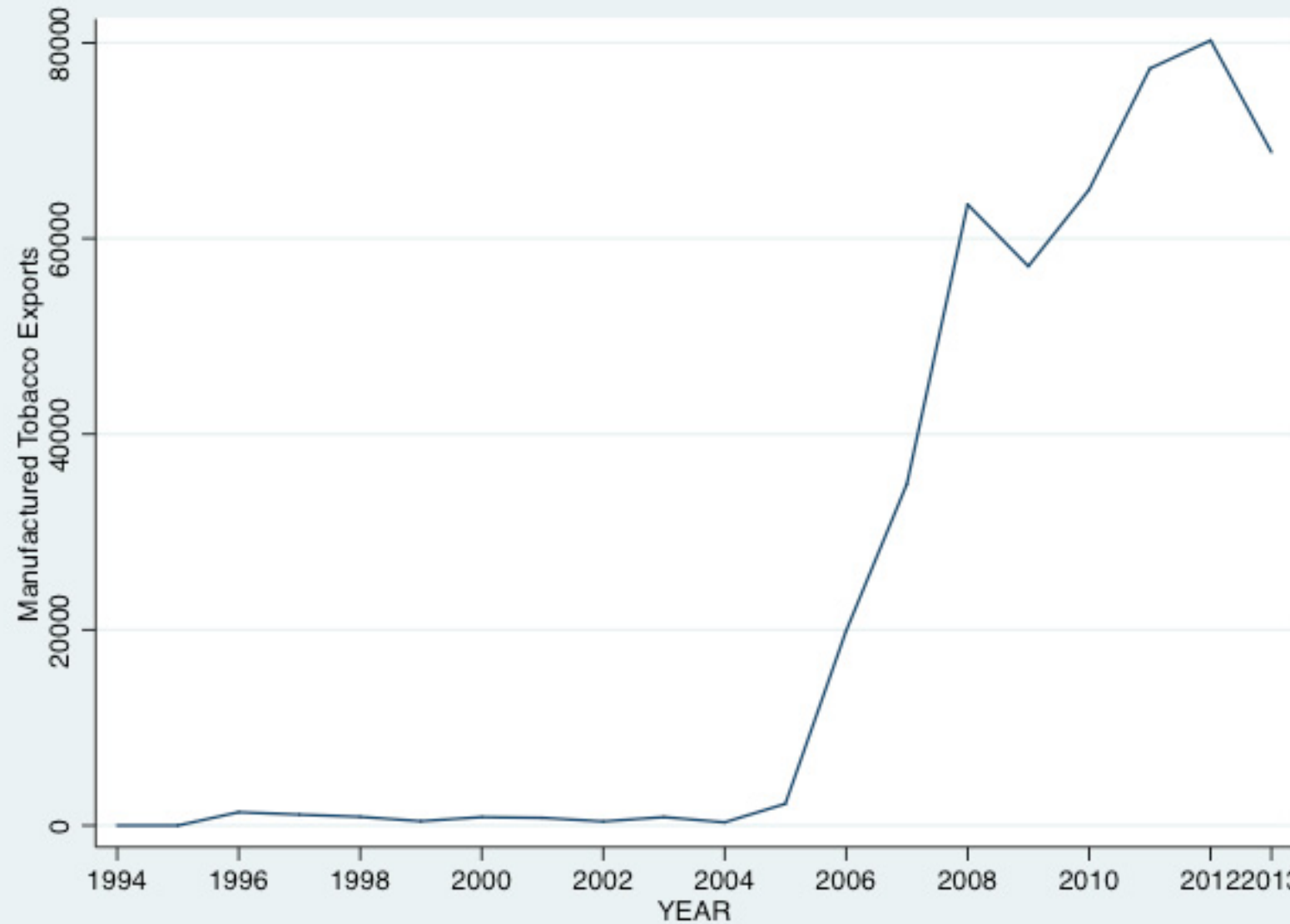

Supplement: Supplementary file 3 — Kenya Manufactured Tobacco Product Exports To Africa (IN 1000 USD). (PDF 125 kb) [file 12992_2017_305_MOESM3_ESM.pdf]

Supplementary Figure 5: Uganda Export Destination

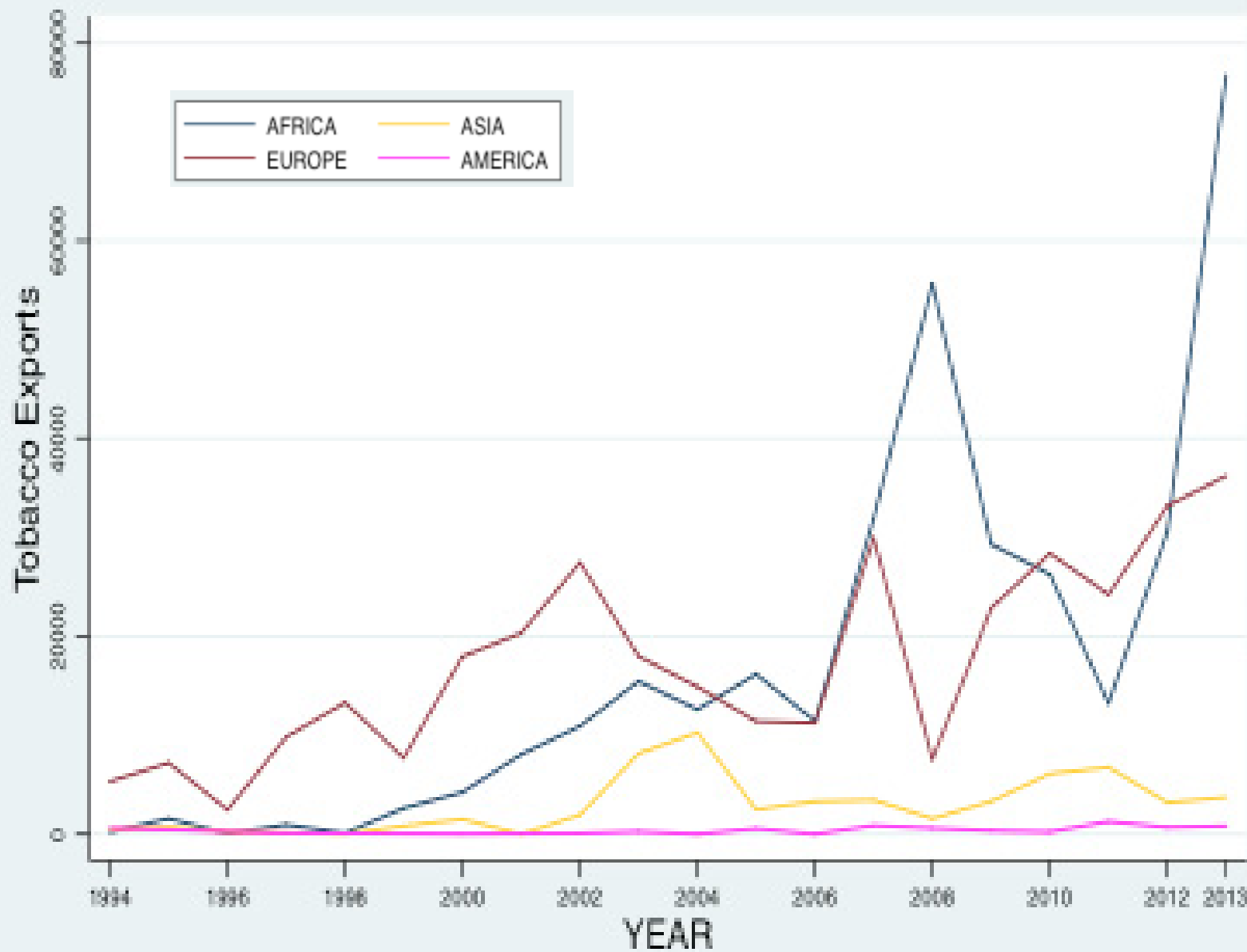

Supplement: Supplementary file 5 — Uganda Export Destination. (PDF 107 kb) [file 12992_2017_305_MOESM5_ESM.pdf]

Supplementary Figure 6: Zambia Export Destination

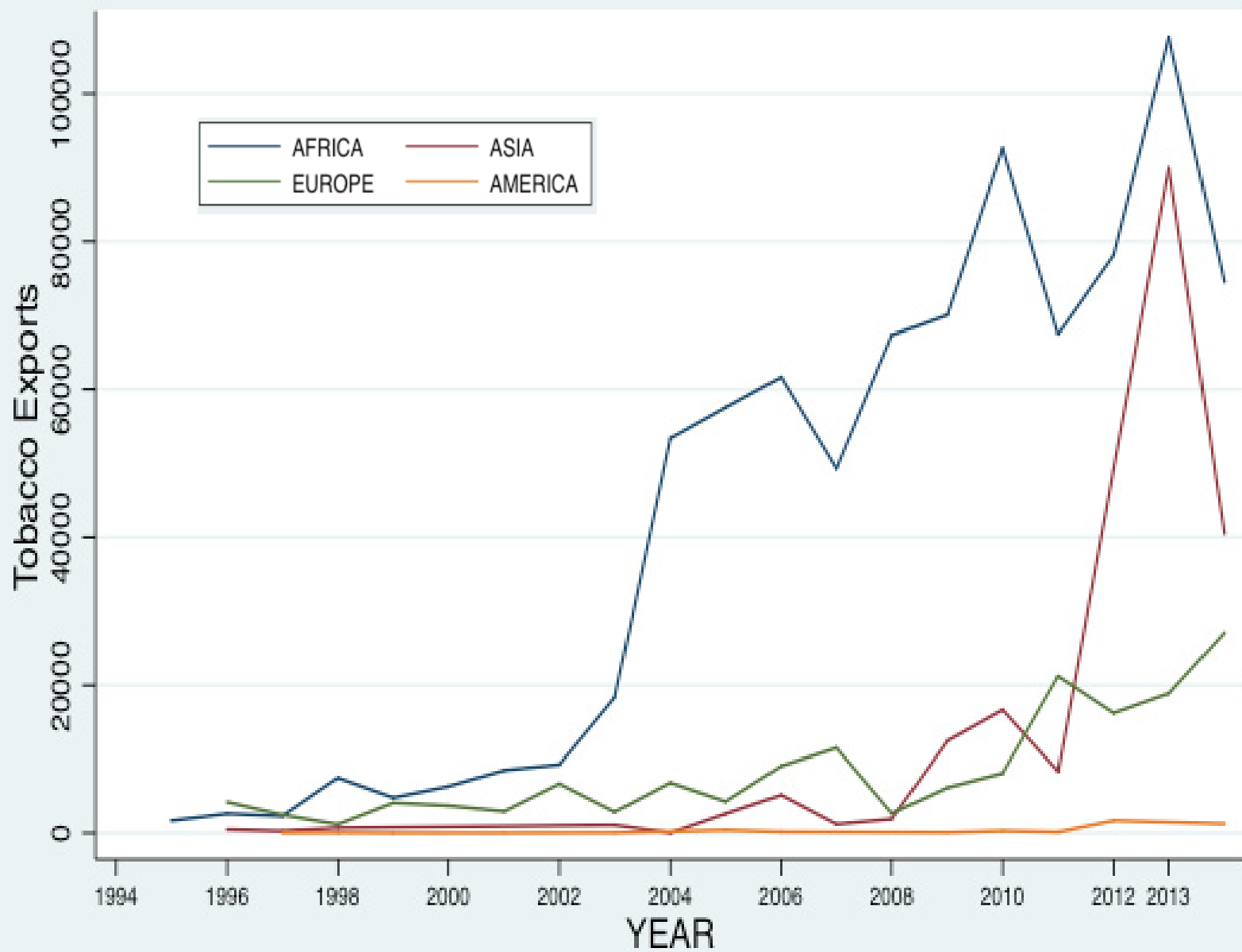

Supplement: Supplementary file 6 — Zambia Export Destination. (PDF 123 kb) [file 12992_2017_305_MOESM6_ESM.pdf]

Supplementary Figure 7: Zimbabwe Export Destination

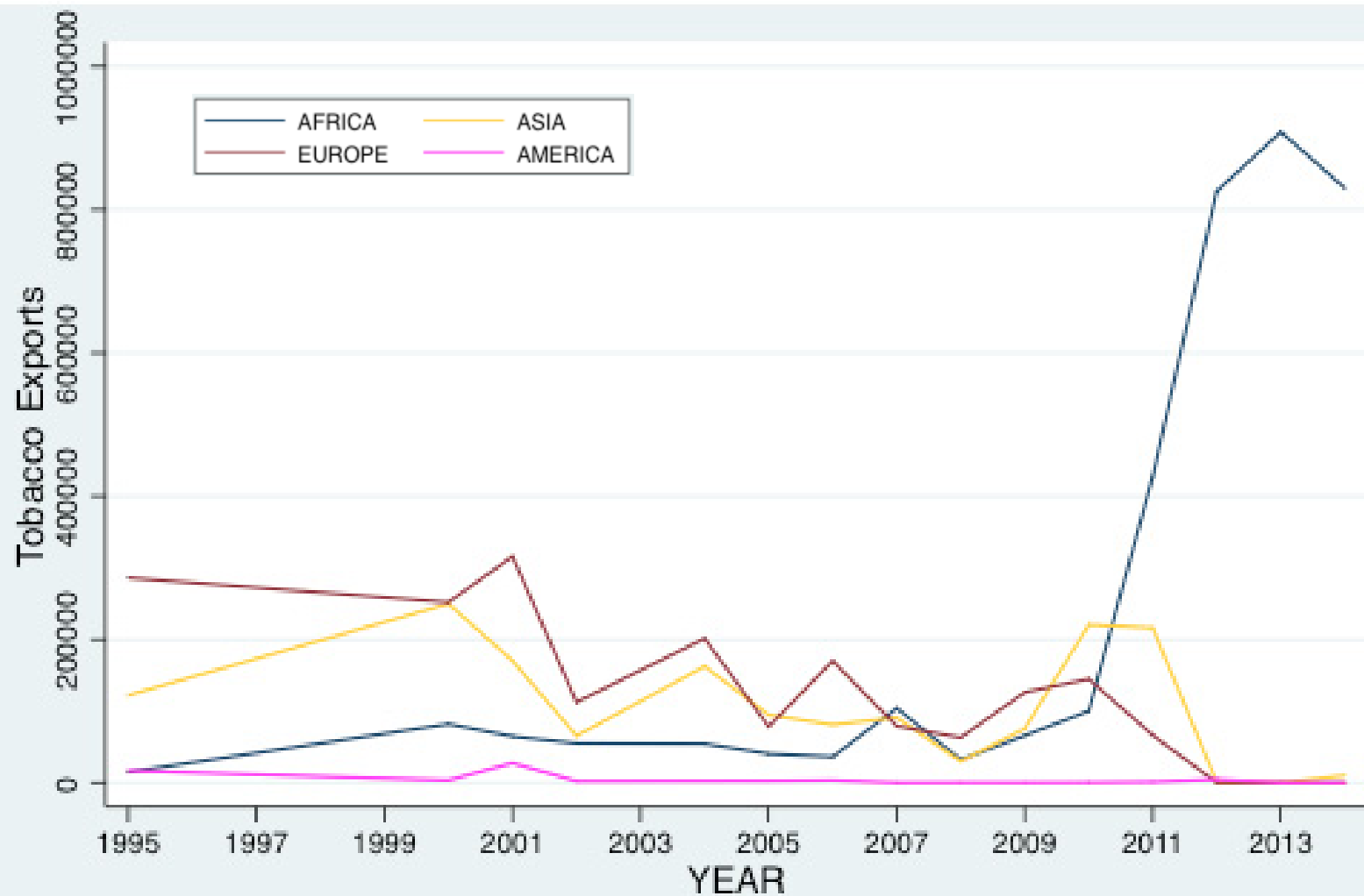

Supplement: Supplementary file 7 — Zimbabwe Export Destination. (PDF 116 kb) [file 12992_2017_305_MOESM7_ESM.pdf]

Supplementary Figure 8: Tanzania Export Destination

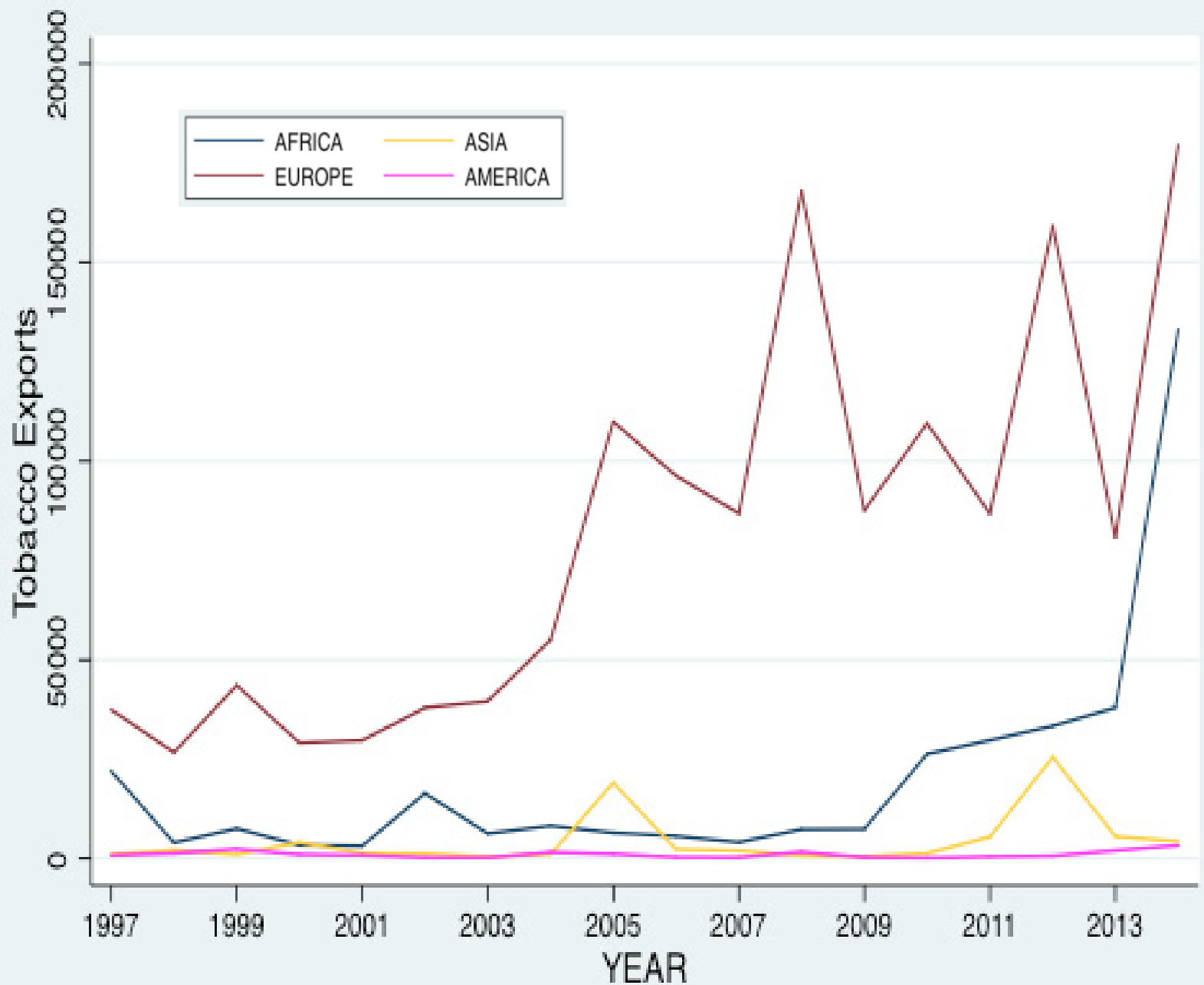

Supplement: Supplementary file 8 — Tanzania Export Destination. (PDF 124 kb) [file 12992_2017_305_MOESM8_ESM.pdf]
