# Supplementary material for: Disentangling regional trade agreements, trade flows and tobacco affordability in sub-Saharan Africa
Source: Global Health. 2017 Nov 14;13:81. doi: 10.1186/s12992-017-0305-x (PMC5686832; doi:10.1186/s12992-017-0305-x)

Supplementary Figure 4: Kenya Manufactured Tobacco Products To Uganda, Rwanda And Mauritius (IN 1000 USD)

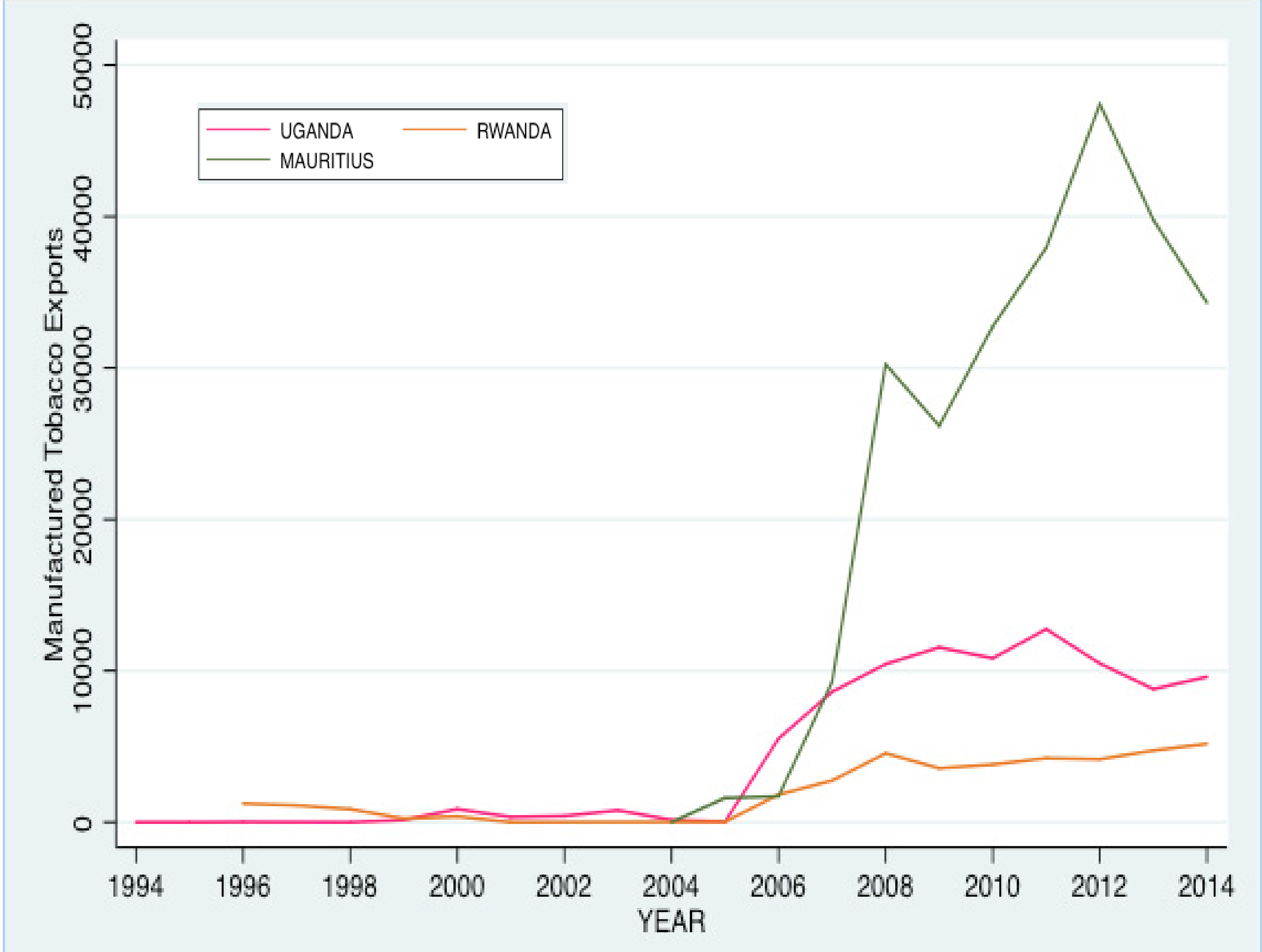

Supplement: Supplementary file 4 — Kenya Manufactured Tobacco Products To Uganda, Rwanda And Mauritius (IN 1000 USD) (PDF 150 kb) [file 12992_2017_305_MOESM4_ESM.pdf]
